# Ablating astrocyte insulin receptors leads to delayed puberty and hypogonadism in mice

**DOI:** 10.1371/journal.pbio.3000189

**Published:** 2019-03-20

**Authors:** Iyad H. Manaserh, Lakshmikanth Chikkamenahalli, Samyuktha Ravi, Prabhatchandra R. Dube, Joshua J. Park, Jennifer W. Hill

**Affiliations:** 1 Department of Physiology and Pharmacology, College of Medicine and Life Sciences, University of Toledo, Toledo, Ohio, United States of America; 2 Center for Diabetes and Endocrine Research, University of Toledo, Toledo, Ohio, United States of America; 3 Department of Neurosciences, College of Medicine and Life Sciences, University of Toledo, Toledo, Ohio, United States of America; UCSD, UNITED STATES

## Abstract

Insulin resistance and obesity are associated with reduced gonadotropin-releasing hormone (GnRH) release and infertility. Mice that lack insulin receptors (IRs) throughout development in both neuronal and non-neuronal brain cells are known to exhibit subfertility due to hypogonadotropic hypogonadism. However, attempts to recapitulate this phenotype by targeting specific neurons have failed. To determine whether astrocytic insulin sensing plays a role in the regulation of fertility, we generated mice lacking IRs in astrocytes (astrocyte-specific insulin receptor deletion [IRKO^GFAP^] mice). IRKO^GFAP^ males and females showed a delay in balanopreputial separation or vaginal opening and first estrous, respectively. In adulthood, IRKO^GFAP^ female mice also exhibited longer, irregular estrus cycles, decreased pregnancy rates, and reduced litter sizes. IRKO^GFAP^ mice show normal sexual behavior but hypothalamic-pituitary-gonadotropin (HPG) axis dysregulation, likely explaining their low fecundity. Histological examination of testes and ovaries showed impaired spermatogenesis and ovarian follicle maturation. Finally, reduced prostaglandin E synthase 2 (PGES2) levels were found in astrocytes isolated from these mice, suggesting a mechanism for low GnRH/luteinizing hormone (LH) secretion. These findings demonstrate that insulin sensing by astrocytes is indispensable for the function of the reproductive axis. Additional work is needed to elucidate the role of astrocytes in the maturation of hypothalamic reproductive circuits.

## Abstract 2

Ablation of insulin receptors in astrocytes reveals that insulin promotes pubertal development and fertility in mice by acting on astrocytes rather than directly affecting neuronal reproductive circuits.

## Author summary

Astrocytes are a major cell type in the central nervous system, yet their impact on the neuroendocrine circuits that control fertility is under appreciated. Here, we show in mice that ablation of insulin signaling in astrocytes leads to delayed puberty, hypothalamic-pituitary-gonadotropin (HPG) axis dysfunction, and reduced fertility. These findings are the first demonstration that astrocytes and a metabolic signal collaborate to permit the maturation of the reproductive axis and adult fertility.

## Introduction

Reproduction is essential for species survival. Because energy is required to locate a mate, maintain a pregnancy, and rear young, fertility is modulated by the status of energy stores [1–3]. Excessive energy expenditure or insufficient caloric intake in humans and rodents delays the pubertal transition and reduces fertility [4, 5]. Moreover, diseases that cause metabolic disturbances, such as thyroid disease, chronic inflammatory states, and malnutrition, are associated with a disruption of the normal timing of puberty [6].

The pancreatic hormone insulin serves as one metabolic signal linking hypothalamic function with metabolic state [7–9]. Postnatal deletion of insulin receptors (IRs) in glial fibrillary acidic protein (GFAP)-expressing cells decreased the activation of pro-opiomelanocortin (POMC) neurons by glucose [10]. Additionally, mice with IR ablated from astrocytes in the mediobasal hypothalamus became insulin and glucose intolerant [10]. These findings suggest that IRs on hypothalamic astrocytes play a role in regulating glucose metabolism.

Insulin is a key regulator of the gonadotropin-releasing hormone (GnRH) network that controls fertility [8, 11–14]. Insulin increases GnRH-dependent luteinizing hormone (LH) secretion in adult male mice [2, 15]. Similarly, hyperinsulinemic clamps in women significantly increase LH pulsatility [2, 16, 17]. Insulin signaling in the brain may also provide a prerequisite signal for the initiation of puberty [18, 19]. Insulin increases in children around the time of adrenarche in association with increasing circulating insulin-like growth factor 1 (IGF1) levels [2]. Administering metformin to girls with precocious pubarche to reduce their insulin levels results in a delay in the onset of puberty [20, 21]. However, the specific mechanisms underlying insulin modulation of pubertal timing are largely unknown.

A seminal paper by Brüning and colleagues [8] showed that 50% of mice lacking the IR in cells expressing nestin (NIRKO mice) displayed hypogonadotropic hypogonadism in adulthood. Targeted deletion of IRs in specific neuronal populations, however, has failed to induce the subfertile phenotype and GnRH network dysregulation of NIRKO mice [2, 3, 6, 22, 23]. For instance, Divall and colleagues found that mice with IR deletion in GnRH neurons experienced normal pubertal timing and fertility [6]. Mice with IR deletion in kisspeptin neurons displayed a 4–5 day delay in pubertal onset but normal fertility and gonadal hormonal levels in adulthood [2]. In another example, mice with IR deletion in gamma-amino butyric acid (GABA)-ergic or glutamatergic cells showed normal pubertal progression, estrous cyclicity, and fertility [23]. More widespread deletion of IR in Ca^2+^/calmodulin-dependent protein kinase-expressing neurons, located in the dentate gyrus, cortex, olfactory bulb, amygdala, striatum, thalamus, and hypothalamus [24], also produced mice with normal reproductive maturation and fertility [3]. These numerous negative results suggest that insulin action in neurons does not play an essential role in hypothalamic-pituitary-gonadal (HPG) axis function.

Alternatively, it has been suggested [3] that the hypothalamic hypogonadism observed in NIRKO mice results from the chronic absence of insulin signaling in glia rather than neurons. Indeed, the nestin-cre line drives deletion of IR in both neuronal and non-neuronal cells [8, 25–27]. Glial cells, which include astrocytes and tanycytes, are known to play an important role in the puberty onset, estrus cyclicity, and fecundity [28, 29]. Therefore, we hypothesized that astrocytic insulin sensitivity is required for normal GnRH release during the pubertal period and in adulthood. We tested this hypothesis by using the cre-lox system to examine the effect of chronic astrocyte IR deletion on fertility.

## Results

### Confirmation of an astrocytic IR knockout model (IRKO^GFAP^)

**Fig 1:**
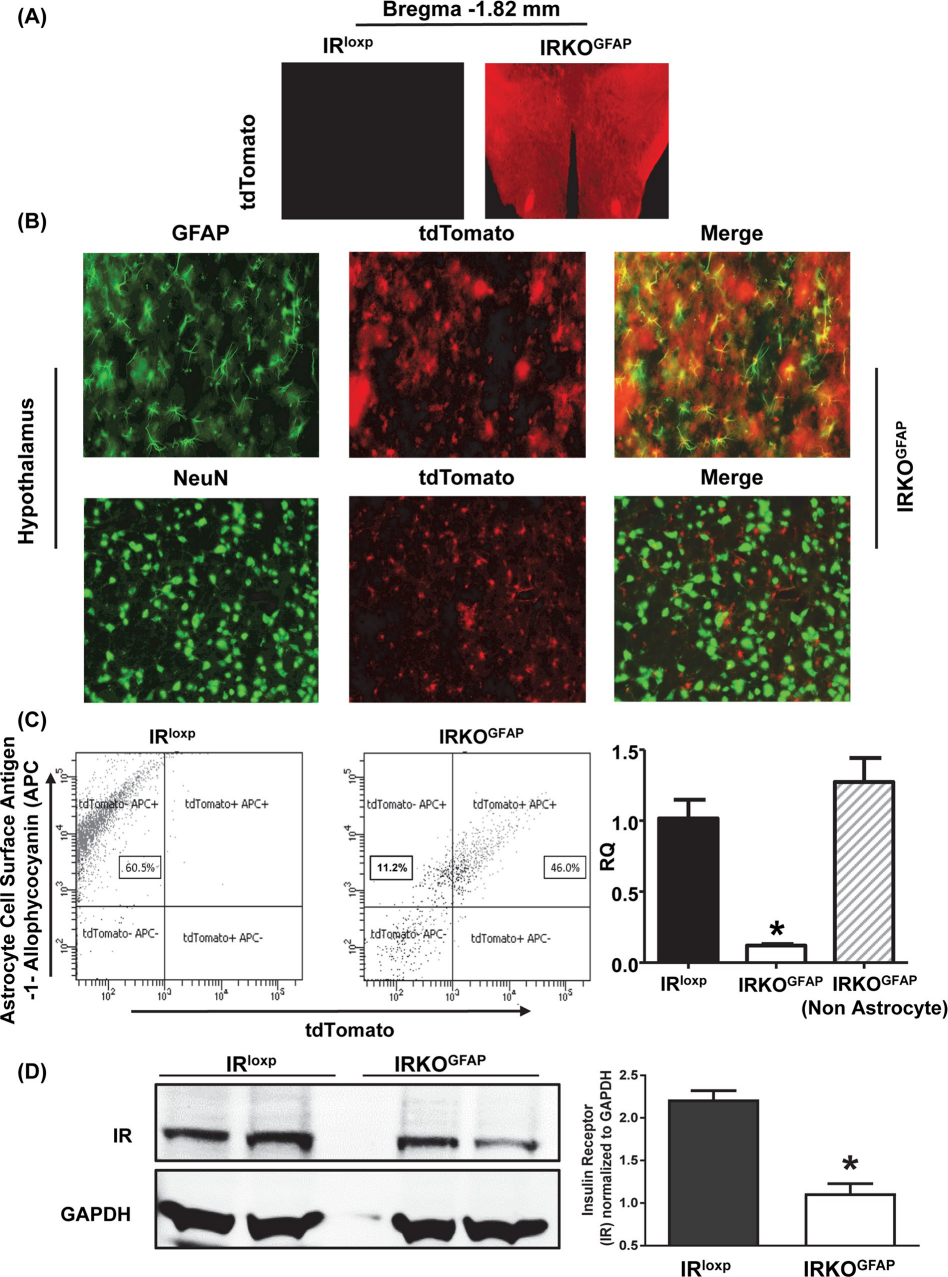
Confirmation of astrocytic IR knockout model (IRKO^GFAP^). (A) Experimental study of cross-section of hypothalamus at Bregma −1.82 mm and Bregma −2.30 mm for IR^loxp^ and IRKO^GFAP^ (*n* = 3–4 per group) using TIRF and confocal microscopy (B) IF cross-sections (50 nm) of hypothalamus for IRKO^GFAP^ stained with GFAP and NeuN antibodies (*n* = 3–4 per group). (C) FACS dot plot showing the sorting gates for tdTomato^low^/APC^low^, tdTomato^low^/APC^high^, tdTomato^high^/APC^low^ and tdTomato^high^/APC^high^ from mice brain hypothalamic cells (*n* = 2 per group). RTPCR of hypothalamic gene expression levels of isolated astrocytes from FACS were reported as relative quantification (RQ = ^2-ΔΔCt^) for IR^loxp^, IRKO^GFAP^ and IRKO^GFAP^ nonastrocyte. IR^loxp^ (black bar), IRKO^GFAP^ (white bar), and IRKO^GFAP^ nonastrocyte (dashed white bar). Values are expressed as means ± SEM. *P < 0.05 IRKO^GFAP^ versus IR^loxp^ group. (D) Western blotting of protein expression from brain tissues were imaged and quantified for IR^loxp^ and IRKO^GFAP^ mice. IR^loxp^ (black bar) and IRKO^GFAP^ (white bar), (*n* = 4 per group, two brains pooled per lane). Values are expressed as means ± SEM. *P < 0.05 IRKO^GFAP^ versus IR^loxp^ group. The underlying data can be found in S1 Data. APC, allopycocyanin; FACS, fluorescence-activated cell sorting; GFAP, glial fibrillary acidic protein; IR; IRKO^GFAP^, astrocyte-specific insulin receptor deletion; RQ, relative quantification.

To generate mice with IR deletion in astrocytes, we crossed IR^loxp^ and GFAP-cre mouse lines. To assess whether Cre expression was restricted to astrocytes in the resulting mice, we crossed experimental mice with tdTomato-loxP reporter mice, which express red fluorescent protein (RFP) in a cre-dependent manner. RFP was found in IRKO^GFAP^ brains but in not those of control mice that carried only the IR^loxp^ allele (Fig 1A). Our data confirm the specificity and selectivity of IR gene and transcript deletion to the brain and not other tissues, including the gonads (S1 Fig). Double immuno-staining labeling of GFAP and tdTomato showed sufficient cre activity to drive tdTomato expression in 94% of GFAP positive cells. When neurons were labeled with the neuronal nuclear antigen NeuN, there was no colocalization with cre-driven tdTomato expression (Fig 1B). We performed immuno-staining colocalization studies in various regions of the brain, including the arcuate nucleus (ARC), anteroventral periventricular nucleus (AVPV), and the cortex to further confirm the wide-spread deletion of IR in astrocytes (S2 Fig).

Fluorescence-activated cell sorting (FACS) was performed on isolated brain cells using tdTomato as a marker of cre expression. The data show that 46.0% of isolated brain cells were positive for astrocyte cell surface antigen-1 (ACSA-1) and tdTomato, whereas 11.2% of cells were positive for ACSA-1 yet negative for tdTomato in the IRKO^GFAP^ mice. In addition, very few cells (0.7%) were positive for tdTomato and negative for ACSA-1 in brain cells isolated from IRKO^GFAP^ mice (Fig 1C). Astrocytes isolated by this method (tdTomato^+^ allopycocyanin^+^ [APC]) showed a substantial reduction in IR mRNA levels in IRKO^GFAP^ mice when compared to IR^loxp^ (tdTomato^−^ APC^+^) (Fig 1C). Meanwhile, the expression levels of IR mRNA in the isolated nonastrocyte cells (tdTomato^−^ APC^−^) from IRKO^GFAP^ mice were comparable to the IR^loxp^ group, confirming the specificity of the deletion (Fig 1C). Previous studies have suggested that tanycytes near the third ventricle express GFAP [30]. Therefore, to further verify the purity of astrocytic FACS isolation, we measured gene expression of different markers of neuronal, tanycytic, microglia, and endothelial markers and confirmed the specific isolation of astrocytes via FACS (S3 Fig). In addition, western blotting of brain tissues confirmed decreased levels of IR protein in IRKO^GFAP^ mice when compared to the IR^loxp^ group (Fig 1D) (S4 Fig). Because it is still unclear if astrocytes are derived from erythromyeloid progenitors, the same lineage that produces macrophages in the periphery [31, 32], we tested whether macrophages, which originate as monocytes produced in bone marrow, exhibited loss of IRs. Expression of IRs and GFAP was not different in macrophages from IR^loxp^ and IRKO^GFAP^ mice (S5 Fig).

### Pubertal timing

**Fig 2:**
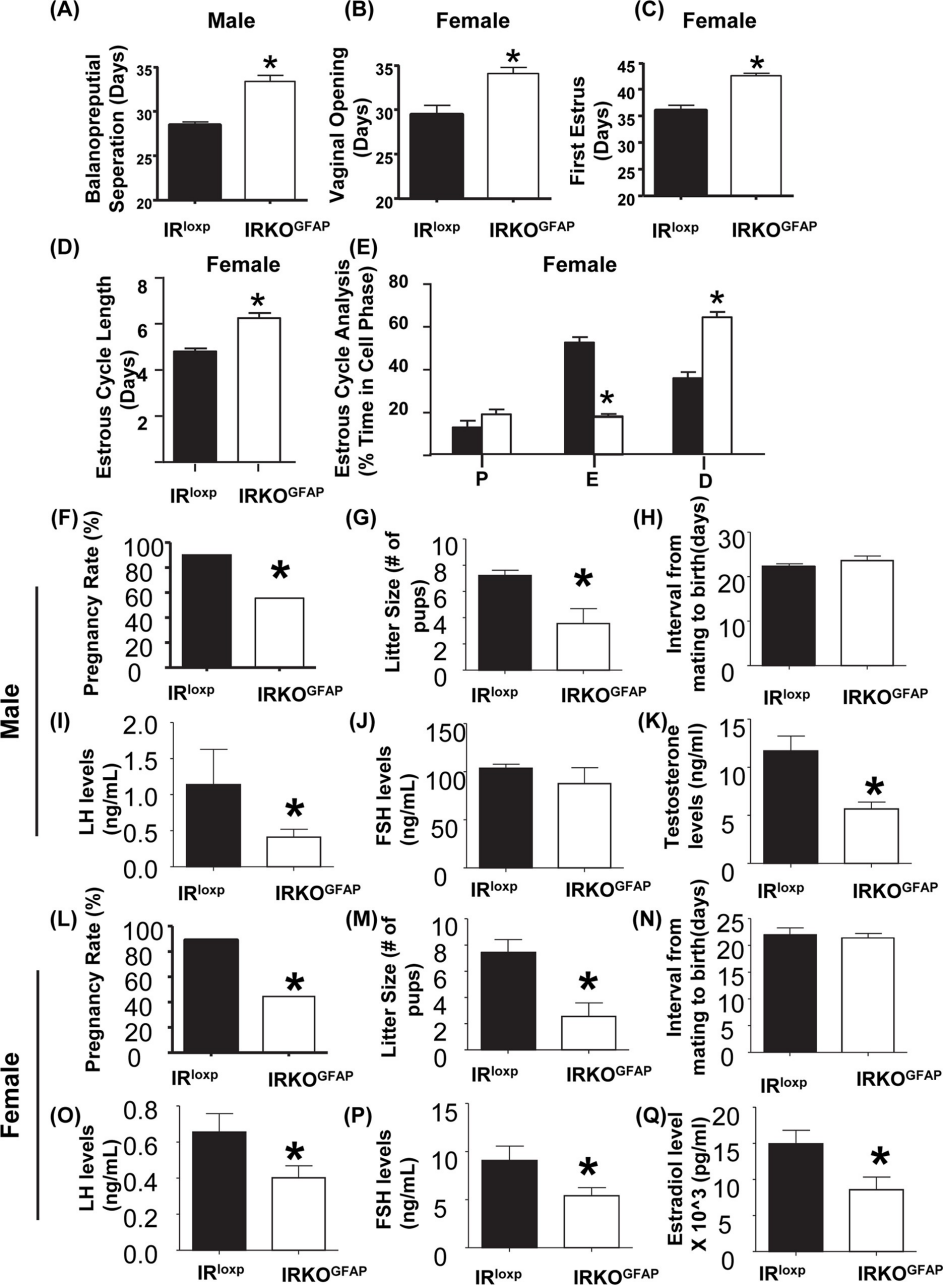
Disruption in pubertal timing and adult fertility. (A–C) Puberty onset was measured as balanopreputial separation in males and vaginal opening and first estrus in females. IR^loxp^ (black bar), IRKO^GFAP^ (white bar), *n* = 10–16 per group. (D) Female adult estrus cycle length and cell type analysis. P (predominantly nucleated cells), E (predominantly cornified epithelium cells), D (predominantly leukocytes), *n* = 10–13 per group. Values are expressed as means ± SEM. *P < 0.05 IRKO^GFAP^ versus IR^loxp^ group. (F–H) Percentage of matings for males resulting in pregnancy. Male litter sizes. Male interval from mating until birth of pups. IR^loxp^ (black bar) and IRKO^GFAP^ (white bar), *n* = 9–10 for males. Values are expressed as means ± SEM. *P < 0.05 IRKO^GFAP^ versus the IR^loxp^ group. (I–K) LH levels of males. FSH level of males. Testosterone levels of males. (*n* = 7–9 per group). IR^loxp^ (black bar) and IRKO^GFAP^ (white bar). Values are expressed as means ± SEM. *P < 0.05 IRKO^GFAP^ versus the IR^loxp^ group. (L–N) Percentage of matings for females resulting in pregnancy. Female litter sizes. Female interval from mating until birth of pups. IR^loxp^ (black bar) and IRKO^GFAP^ (white bar), *n* = 9 for females. Values are expressed as means ± SEM. *P < 0.05 IRKO^GFAP^ versus the IR^loxp^ group. (O–Q) LH levels of females. FSH level of females. Estradiol level of females. (*n* = 6–8 per group). IR^loxp^ (black bar) and IRKO^GFAP^ (white bar). Values are expressed as means ± SEM. *P < 0.05 IRKO^GFAP^ versus the IR^loxp^ group. The underlying data can be found in S1 Data. D, diestrus; E, estrus; FSH, follicle-stimulating hormone; IR, insulin receptor; IRKO^GFAP^, astrocyte-specific insulin receptor deletion; LH, luteinizing hormone; P, proestrus.

Balanopreputial separation serves as an indicator of the initiation of puberty in males. IRKO^GFAP^ male mice showed a significant delay in the postnatal day (PND) of balanopreputial separation (PND 33.36 ± 0.67) when compared to IR^loxp^ control mice (PND 28.44 ± 0.36) (Fig 2A). In contrast, we found that the GFAP-cre mouse line alone has no phenotype in comparison to IR^loxp^ mice (S6 Fig).

To assess the progression of puberty in female mice, vaginal opening and timing of the onset of estrus cycling were measured. IRKO^GFAP^ mice exhibited a delay in vaginal opening of approximately 4 days (PND 34.08 ± 0.69) when compared to IR^loxp^ mice (PND 29.44 ± 1.05) (Fig 2B). IRKO^GFAP^ mice showed a significant delay in the age of first estrus by approximately 5 days (PND 42.55 ± 0.45) when compared to IR^loxp^ mice (PND 36.00 ± 1.01) (Fig 2C). In addition, no differences were seen in body weight or body growth at 3 weeks of age between IRKO^GFAP^ and IR^loxp^ mice (S7 Fig).

### Adult fertility

IRKO^GFAP^ females exhibited irregular cyclicity and longer estrous cycles. The estrus cycle length was approximately 2 days longer in IRKO^GFAP^ females (PND 6.25 ± 0.21) when compared to IR^loxp^ mice (PND 4.80 ± 0.13) (Fig 2D). IRKO^GFAP^ mice spent significantly less time in estrus and a longer time in diestrus when compared to IR^loxp^ females (Fig 2E) (S8 Fig).

To assess fertility in IRKO^GFAP^ mice, pregnancy rate, litter size, and mating success were measured. IRKO^GFAP^ males produced fewer pregnancies when paired with fertile wild-type (WT) females (54% induced pregnancies), while IR^loxp^ males were 90% successful in producing pregnancies (Fig 2F). IRKO^GFAP^ females, when paired with fertile WT males, exhibited a significantly reduced pregnancy rate of 45%, compared to 89% for IR^loxp^ females (Fig 2L). The interval from mating to birth did not differ between groups (Fig 2H and 2N). However, IRKO^GFAP^ male and female mice exhibited a significant decrease in litter size when compared to IR^loxp^ mice (litter size for IR^loxp^ 7.44 ± 0.97 versus IRKO^GFAP^ 2.55 ± 1.02) (Fig 2G and 2M).

### Hormonal and gonadal assessments

We next assessed the function of the HPG axis in adult male and randomly cycling female mice by measuring LH, follicle-stimulating hormone (FSH), and sex steroid levels between 8 and 10 AM. IRKO^GFAP^ males showed a significant decrease in LH and testosterone levels (Fig 2I and 2K) but no change in FSH when compared to IR^loxp^ mice (Fig 2J). LH, FSH, and estradiol levels were significantly decreased in IRKO^GFAP^ females when compared to IR^loxp^ mice (Fig 2O–2Q). LH pulse amplitude and frequency have been reported to be reduced on estrus, although basal levels of LH are similar on all days of the cycle [33]. Since IRKO^GFAP^ female mice spent less time in estrus yet had lower LH levels, mouse cycle stage is unlikely to explain these findings.

**Fig 3:**
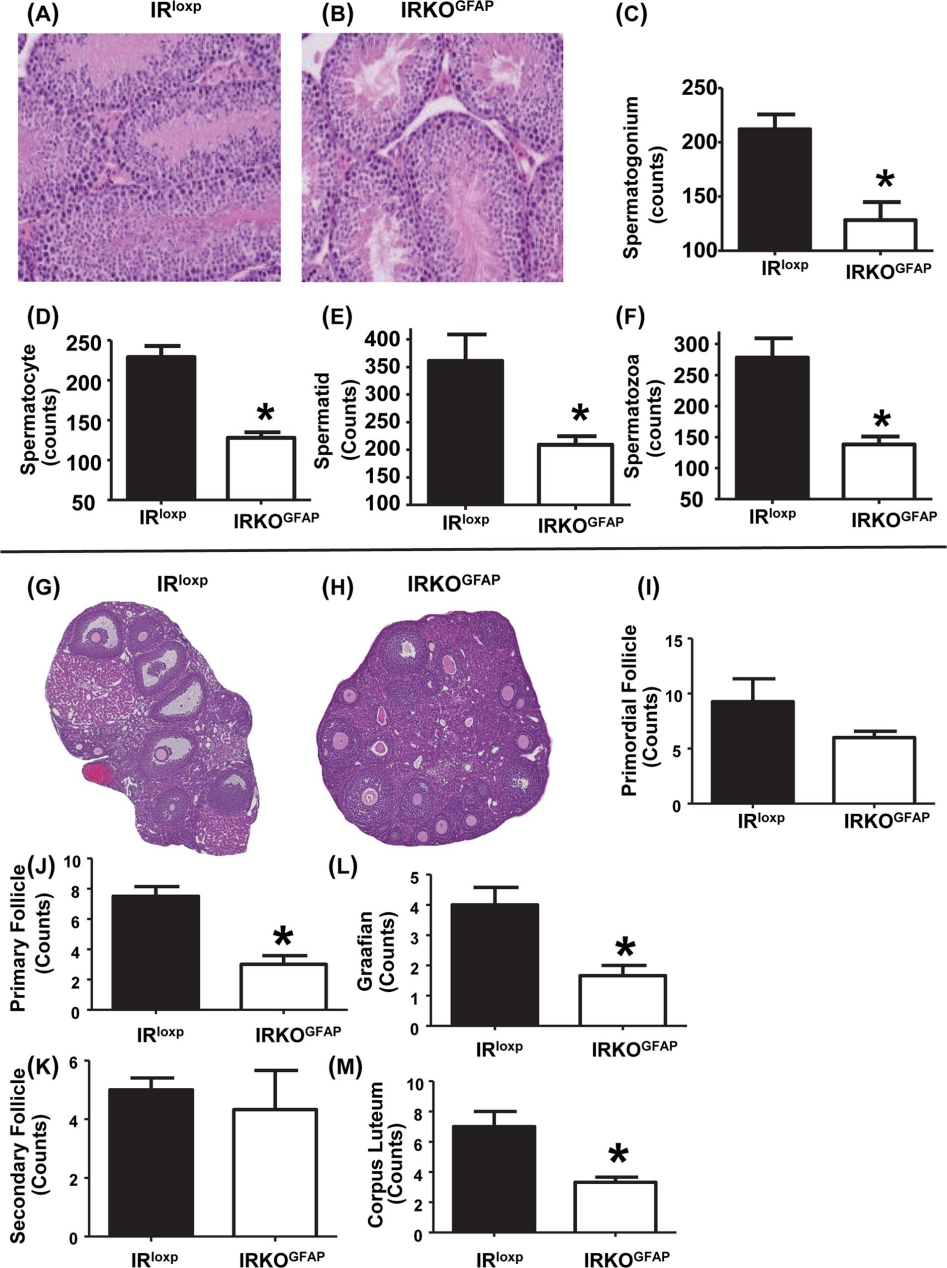
Altered testes morphology and impaired spermatogenesis as well as ovarian morphology and follicle maturation at 6–7 months of age. (A–B) Histological images of representative IR^loxp^ and IRKO^GFAP^ testes. (C–F) Analysis of number of spermatogonium, spermatocyte, spermatid, and spermatozoa of IR^loxp^ (*n* = 5) and IRKO^GFAP^ (*n* = 4). IR^loxp^ (black bar) and IRKO^GFAP^ (white bar). Values are expressed as means ± SEM. *P < 0.05 IRKO^GFAP^ versus the IR^loxp^ group. Histological images of IR^loxp^ (*n* = 4) and IRKO^GFAP^ (*n* = 3) female mice IR^loxp^ (black bar) and IRKO^GFAP^ (white bar). (I–M) Ovarian follicle maturation analysis of different follicle stages (primordial, primary, secondary, and Graafian) and corpora lutea in IR^loxp^ (*n* = 4) and IRKO^GFAP^ (*n* = 3) mouse ovaries. Values are expressed as means ± SEM. *P < 0.05 IRKO^GFAP^ versus the IR^loxp^ group. The underlying data can be found in S1 Data. IR, insulin receptor; IRKO^GFAP^, astrocyte-specific insulin receptor deletion.

Gonadal morphology was examined in both sexes. There was a reduction in the sperm count per seminiferous tubule cross-section in all stages (Fig 3A and 3B). Spermatogonia, spermatocytes, spermatid, and spermatozoa counts were significantly reduced in the seminiferous tubules of IRKO^GFAP^ males (128.3 ± 16.53, 128.0 ± 7.16, 209.0 ± 15.76, and 138.3 ± 12.61) when compared to IR^loxp^ mice (212.0 ± 13.72, 229.0 ± 14.01, 361.0 ± 48.30, and 278.0 ± 31.10) (Fig 3C–3F). IRKO^GFAP^ female mice exhibited altered ovarian morphology when compared to IR^loxp^ mice (Fig 3G and 3H). Similarly, the number of primary follicles, preovulatory follicles, and corpora lutea per ovary cross-section were significantly lower (3.00 ± 0.57, 1.67 ± 0.33, and 3.33 ± 0.33) when compared to IR^loxp^ mice (7.50 ± 0.64, 4.00 ± 0.57, and 7.00 ± 1.00) (Fig 3I–3M). Primordial and secondary follicle numbers were not different between groups.

### Sexual behavior

Because astrocytic insulin signaling has been linked to depressive-like behavior [69], we examined sexual behavior in these mice to determine whether reduced fertility in IRKO^GFAP^ mice could be partially attributed to reduced sexual motivation or performance. IRKO^GFAP^ and IR^loxp^ females were paired with WT gonadectomized males, and multiple parameters were measured, including lordosis, mounting attempts, lordosis quotient, and latency to first lordosis. IRKO^GFAP^ and IR^loxp^ female mice showed no differences in any of these parameters (S9 Fig). Likewise, IRKO^GFAP^ and IR^loxp^ male mice showed no differences in mounting attempts, latency to first mount, and latency to first intromission when paired with control females (S9 Fig).

### Astrocyte prostaglandin E2 synthesis

**Fig 4:**
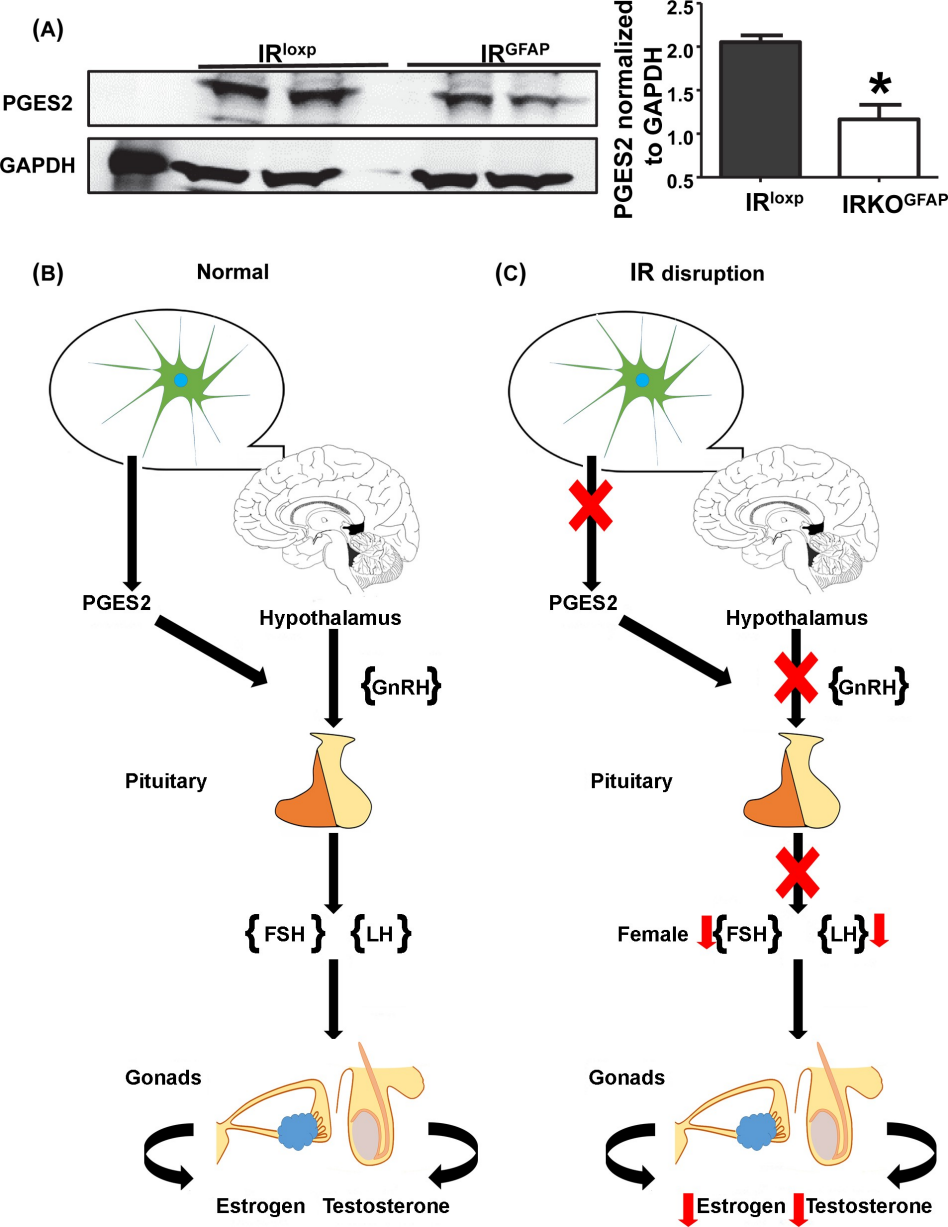
Altered PGES2 levels. (A) Western blotting of PGES2 protein expression from FACS-isolated astrocytes were imaged and quantified for IR^loxp^ and IRKO^GFAP^ mice. IR^loxp^ (black bar) and IRKO^GFAP^ (white bar), (*n* = 4 per group, 2 brains pooled per lane). Values are expressed as means ± SEM. *P < 0.05 IRKO^GFAP^ versus IR^loxp^ group. The underlying data can be found in S1 Data. (B–C) Schematic diagram representing the mechanism of astrocyte modulation of HPG axis. FACS, fluorescence-activated cell sorting; HPG, hypothalamic pituitary gonadotropin; IR, insulin receptor; IRKO^GFAP^, astrocyte-specific insulin receptor deletion; PGES2, prostaglandin E synthase 2.

Astrocytes release specific growth factors that stimulate the secretion of GnRH. In particular, prostaglandin E2 (PGE2) release stimulates the secretion of GnRH; Clasadonte and coworkers investigated the firing activity of GnRH neurons in mice with deficient PGE2 synthesis in astrocytes and found the excitability of these neurons significantly decreased [34]. We therefore measured protein levels of prostaglandin E synthase 2 (PGES2), which catalyzes the conversion of prostaglandin H2 to prostaglandin E2, in isolated astrocytes from IRKO^GFAP^ and control mice. IRKO^GFAP^ astrocytes exhibited a significant reduction in PGES2 levels when compared to IR^loxp^ astrocytes (Fig 4A–4C).

## Discussion

Astrocytes assist neurons through nutritional and structural support and by promoting neurotransmitter release and recycling. They also appear to contribute to information processing by the brain [35, 36]. Astrocytes possess a dense network of fine processes whose membranes contain potassium channels [37, 38], aquaporins [39], glutamate transporters [40], and lactate transporters [41]. These processes enwrap neuronal synapses and ensure effective synaptic transmission. Astrocytes also display increased intracellular calcium (but not electrical excitability) in response to chemical and neuronal cues [42], which is believed to lead to the release of gliotransmitters, such as adenosine, polyphosphate, D-serine, glutamate, GABA, and lactate, that can alter neuronal activity [43–48]. As one critical element of the blood–brain barrier, astrocytes are readily able to sense circulating metabolic and endocrine signals [49, 50]. Notably, insulin acts on IRs in primary human astrocytes, promoting glycogen synthesis [51]. Astrocytes are also able to release vasoactive molecules to regulate cerebral blood flow and to ensure a sufficient supply of oxygen and glucose to active neurons [52]. Astrocytes are therefore believed to play a critical role as central nervous system (CNS) metabolic sensors [53].

The current study demonstrates that insulin is a critical metabolic signal acting through astrocytes to permit reproductive competency via the GnRH network; astrocyte insulin signaling prevented hypogonadism and allowed normal fertility in adulthood. Similar to NIRKO mice [54], IRKO^GFAP^ mice exhibited impaired spermatogenesis, folliculogenesis, and ovulation, resulting in an almost 50% decrease in pregnancy rate and a nearly 69% reduction in litter size. IRKO^GFAP^ mice also showed a significant decrease in LH and testosterone levels in males and LH, FSH, and estradiol levels in females. These findings indicate that disruption of astrocytic insulin signaling leads to hypogonadotropic hypogonadism [55, 56]. Given that IRKO^GFAP^ mice exhibit a delay in vaginal opening and first estrous in females and balanopreputial separation in males, disruption of astrocytic insulin action also serves as a critical role in the maturation of the HPG axis.

Astrocytes have the potential to control GnRH release in several ways. GFAP-immunoreactive astrocyte processes have been shown to ensheath GnRH cell bodies in the rostral preoptic area of the rat [57] and GnRH cell bodies in the medial basal hypothalamus of monkeys [58, 59]. In addition, GnRH processes in the median eminence are apposed largely by astrocytes, with the support of tanycytes [60]. The structural relationships at both sites are dynamic and regulated by gonadal steroids in rodents and rhesus monkeys [57, 58, 61, 62]. GnRH neurons adhere to astrocytes using heterophilic (contactin/RPTPβ) and homophilic synaptic cell adhesion molecule (SynCAM) interactions; these molecules have signaling capabilities, suggesting they can activate intracellular signaling cascades in astrocyte and GnRH neurons [63]. Indeed, transgenic mice that express a dominant negative SynCAM1 under the control of a human GFAP promoter had a delayed onset of puberty, disrupted estrous cyclicity, and reduced fecundity associated with low GnRH release [29].

Astrocytes also synthesize and release factors that regulate GnRH secretion [28]. Astrocytes are believed to produce growth factors such as basic fibroblast growth factor IGF1 and transforming growth factor (TGF)-β1 that act directly on GnRH neurons to stimulate production of GnRH. In addition, in vitro evidence suggests that their production of growth factors of the epidermal growth factor family (TGFα and neuroregulins) causes glial release of mediators like PGE2 that stimulate GnRH release [64]. Mice expressing a dominant-negative Erbb2 receptor tyrosine kinase 4 receptor, which responds to EGFs, under the control of the GFAP promoter exhibit delayed sexual maturation and a diminished reproductive capacity in early adulthood due to impaired release of GnRH [65]. Interestingly, human hypothalamic hamartomas associated with sexual precocity in humans contain numerous astrocytes expressing TGFα and erbB1 receptors [66].

Astrocytes also release substances, like calcium, glutamate, and ATP, capable of stimulating GnRH release [67, 68]. Cai and coworkers (2018) recently found that insulin signaling can target astrocyte-specific soluble NSF attachment protein receptors to regulate exocytosis of ATP [69]. Thus, IR deletion in IRKO^GFAP^ mice may lead to impaired tyrosine phosphorylation of mammalian uncoordinated-18, leading to decreased astrocytic ATP exocytosis [69]. Finally, neurons require glial-provided precursors such as glutamine to synthesize glutamate and GABA. This mechanism allows astrocytes to influence neuronal glutamate production and availability at the synaptic cleft by expressing glutamine synthase [70, 71]. This regulation is responsive to estradiol levels and pubertal progression [72, 73]. Overall, these studies demonstrate that astrocytes can influence GnRH release through multiple pathways.

Studies have shown that hypothalamic astrocytes release PGE2 in response to cell–cell signaling. PGE2 release stimulates the secretion of GnRH to regulate the pituitary release of LH and FSH [34]. Our work shows decreased levels of astrocytic PGES2 protein levels in knockout mice when compared to controls, suggesting reduced production and release of PGE2. Interestingly, PGE2 release is mediated by exocytosis. Shimada and colleagues have shown that solute carrier organic anion transporter family member 2A1, a PGE2 transporter, is responsible for loading intracellular PGE2 into lysosomes in macrophages; PGE2 is then released via exocytosis induced by Ca^2+^ influx [74]. Future studies should therefore investigate whether impaired insulin-dependent exocytosis could also affect PGE2 release from astrocytes. Another important consideration for future study is the role of astrocyte insulin action during development versus its actions in the adult animal. Indeed, insulin and IGFs may directly influence brain development and neuronal survival [75–77]. While the contribution of astrocyte insulin signaling to the establishment of neuroendocrine function is unknown, it may play a role during the organization of reproductive circuitry.

In summary, our findings suggest that impaired insulin sensing in astrocytes delays the initiation of puberty and dramatically reduces adult reproductive success. These effects are due to dysfunction of the HPG axis, leading to hypogonadotropic hypogonadism, and are associated with decreased PGES2 levels in astrocytes. This model is the first to recapitulate the effects of brain IR deletion on fertility. Our findings emphasize the importance of astrocytic signaling in the regulation of reproduction and lay the foundation for future studies addressing this communication at different stages of development. Additional studies are warranted to investigate the mechanism of how insulin action on astrocytes modulates the GnRH network.

## Material and methods

### Ethics statement

All procedures were approved by the Institutional Animal Care and Use Committee (IACUC) of the University of Toledo College of Medicine and Life Sciences in Toledo, Ohio. All experiments were performed in accordance with the relevant guidelines and regulations described in the IACUC-approved protocol number 106448.

### Animal and genotyping

To create an astrocyte-specific deletion of IR (IRKO^GFAP^ mice), GFAP-Cre mice (C57Bl/J6) (Frederick National Laboratory for Cancer Research, Frederick, Maryland, United States) were crossed with IR^loxp^ mice (C57Bl/J6) in which exon 4 of the IR gene was flanked by loxP sites [22]. GFAP is the main intermediate filament protein in mature astrocytes and an important component of the cytoskeleton in astrocytes during development [78, 79]. After the first generation of the breeding, GFAP-Cre, IR^loxp^ mice were crossed with homozygous IR^loxp^ mice to generate the experimental mice. IR^loxp^ mice littermates lacking Cre expression were used as controls; comparisons between IR^loxp^ mice and GFAP-Cre mice were also performed where specified. Where noted, the mice also carried the tdTomato gene inserted into the Gt(ROSA)26Sor locus to serve as a reporter under the control of Cre recombinase expression. Mice were housed in the University of Toledo College of Medicine animal facility at 22°C–24°C on a 12-hour light/dark cycle and were fed standard rodent chow. Mice were weaned on postnatal day (PND) 21. Genotyping was performed by Transnetyx, Inc. (Cordova, Tennessee, US) using a real-time RTPCR–based approach.

### Quantitative real-time RTPCR for brain and bone marrow–derived macrophages (BMDMs)

Mice were sacrificed via ketamine/xylazine injections, and the brain and other tissues were removed. Total RNA was extracted using an RNeasy Lipid Tissue Mini Kit (Qiagen, Valencia, California, US). Single-strand cDNA was synthesized by a high-capacity cDNA Reverse Transcription Kit (Applied Biosystems). Bone marrow–derived macrophages were obtained, as previously described [80]. Specifically, femurs and tibias were collected and flushed with medium containing sterile RPMI, 1% penicillin/streptomycin, and L929‐conditioned medium to isolate bone marrow cells. These cells were then allowed to differentiate for 7 days (37°C, 5% CO_2_ atmosphere) with a change of media on day 4. Then, RTPCR was performed [81]. Briefly, total RNA was prepared from BMDMs using Perfect Pure RNA Tissue kit (5Prime kit) according to manufacturer's instructions. cDNA was synthesized with random primers and reverse transcriptase (Applied Biosystems) using 1 μg of total RNA. cDNA was evaluated with quantitative RTPCR using True Amp SYBR green qPCR Supermix (Applied Biosystems). The relative amount of mRNA was calculated by comparison to the corresponding controls and normalized relative to Glyceraldehyde 3-phosphate dehydrogenase (GAPDH). RQ is expressed as means ± SE relative to IR^loxp^. Sequences of primers used are as follows: IR: Forward—CCCCAACGTCTCCTCTACCA, Reverse—TGTTCACCACTTTCTCAAATG; GFAP: Forward—ACATCGAGATCGCCACCTAC, Reverse—ATGGTGATGCGGTTTTCTTC; CD68: Forward—TCCAAGCCCAAATTCAAATC, Reverse—ATATGCCCCAAGCCTTTCTT; MAP-1: Forward—AGTGAGAAGAAAGTTGCCATCATC, Reverse—TTAATAAGCCGAAGCTGCTTAGG; CD11b: Forward—TGCCAAGACGATCTCAGCAT, Reverse—GCCTCCCACCACCAAAGT; Hes-1: Forward—CAACACGACACCGGACAAAC, Reverse—GTGGGCTAGGGACTTTACGG; Hes-5: Forward—GGTACAGTTCCTGACCCTGC, Reverse—AGAGGGTGGGCCCTGATTAT; vWF: Forward—CTACCTAGAACGCGAGGCTG, Reverse -CATCGATTCTGGCCGCAAAG; GAPDH: Forward—CCAGGTTGTCTCCTGCGACT, Reverse—ATACCAGGAAATGAGCTTGACAAAGT.

### FACS

Mice were sacrificed via ketamine/xylazine injections, and brains were collected. The hypothalami were then excised and minced with a razor blade on an ice-cold glass plate and placed in a microfuge tube with 1 ml of hibernate A (HA-LF; Brian Bits, Springfield, Illinois, US). Hibernate A was then replaced with 1 ml Accutase (SCR005, Millipore, Temecula, California, US), and tubes were rotated for 30 minutes at 4°C. Samples were centrifuged at 425 x g for 2 minutes and each pellet was resuspended in 250 μl of ice-cold Hibernate A [82]. For cell dissociation, samples were triturated 10 times with a large Pasteur pipet and then placed on ice. Large pieces were allowed to settle, and 600 μl of supernatant was transferred to a 15-ml Falcon tube on ice. 600 μl of Hibernate A was added to the original tube, and the same procedure was repeated with medium and small Pasteur pipets. The collected supernatants were transferred to a 15-ml Falcon tube. Lastly, 750 μl of Hibernate A was added to the original tube, and 800 μl of supernatant was added to the 15-ml Falcon tube. Large debris was removed from the cell suspension by serial filtration through 100-μm and 40-μm cell strainers into 50-ml Falcon tubes, respectively (Falcon 352360; Falcon 352340; BD Biosciences, San Jose, California) [82]. The cell suspension was then centrifuged at 300 x g for 10 minutes and supernatant was aspirated completely. 100 μl of buffer (PBS +5% FBS) per 10^6^ nucleated cells was added to the pellet. Then, 10 μl of ACSA-1 antibody (MACS Cat. #130-095-814) was added, mixed well, and incubated for 10 minutes in the dark. Cells were washed by adding 1 ml of buffer and centrifuged at 300 x g for 10 minutes. The supernatant was then aspirated completely. Lastly, the cell pellet was resuspended in 500 μl of buffer. Cells were sorted in FACSAria (BD Biosciencs, San Jose, California) using tdTomato and ACSA-1-APC appropriate wavelengths (581 nm and 660 nm, respectively) [83]. Astrocytes were isolated from IR^loxp^ (tdTomato^−^ APC^+^), and IRKO^GFAP^ (tdTomato^+^ APC^+^). In addition, nonastrocyte cells were isolated from IRKO^GFAP^ (tdTomato^−^ APC^−^/ tdTomato^+^ APC^−^/ tdTomato^−^ APC^+^). RNA from these cells were purified to determine IR gene expression [84].

### Magnetic cell sorting and western blotting

Mice were sacrificed via ketamine/xylazine injections, and brains were collected, then excised and minced with a razor blade on an ice-cold glass plate and placed in a microfuge tube with 1 ml of hibernate A (HA-LF; Brian Bits, Springfield, Illinois). A similar procedure was followed to isolate brain cells, as previously described in the FACS method section. Then, astrocytes expressing NA^+^-dependent glutamate transporter (GLT-1) were positively selected using rabbit anti GLT-1 antibody (Cat. #OSE0004W, ThermoFisher Sci) and goat antirabbit IgG magnetic beads (Cat. #S1432S, Biolabs). Full details of the procedure were described previously [85]. For protein expression, isolated astrocytes were lysed in RIPA buffer (Cat. #SC-24948, Santa Cruz Biotech). Lysate was centrifuged, followed by BCA assay to determine protein concentration. The primary antibodies used were as follows: IRβ (Cat. #3025S, Cell signaling); PGES2 (Cat. #bs-2639R, Bioss) [86, 87]; and GADPH (Cat.# SC-32233, Santa Cruz Biotechnology). Secondary antibodies used were as follows: goat antirabbit-800 (LI-COR, P/N 925–32211) and donkey antimouse-680 (LI-COR, P/N 925–68075). Images were captured using the LI-COR odyssey infrared imaging system, and only the contrast and brightness were adjusted for this purpose.

### Perfusion and immunofluorescence

Adult males and females (in diestrus) were perfused at the age of 7–8 months. Brains of the mice were collected and postfixed with 10% formalin at 4°C overnight, followed by immersion in 10%, 20%, and 30% sucrose for 24 hours each. A sliding microtome was used to cut sections (35–40 μm) of the brain into five series [2, 88]. For immunofluorescence, these sections were permeablized in 1 x PBS / 0.4% Triton x 100 for 1 hour at room temperature. Then, they were blocked in 1% BSA/5% normal donkey serum in 1 x PBS/Triton 0.4% at room temperature for 1 hour. After that, tissues were incubated with primary antibodies in blocking buffer at 4°C overnight, followed by five washes in PBST, with each wash lasting 10 minutes. Then, the tissues were incubated with secondary antibodies in blocking buffer for 2 hours at room temperature, followed by five washes in PBST. Sections were mounted on slides, air-dried overnight, and coverslipped with fluorescence mounting medium containing DAPI (Vectasheild, Vector laboratories, Inc. Burlingame, California). Brain sections were visualized for the expression of tdTomato, GFAP, and NeuN fluorescence in IRKO^GFAP^ mice using Total Internal Reflection Microscopy (B&B microscopy limited Olympus IX-81) and Confocal Microscopy (Leica) and captured via Metaphore for Olympus Premier software. The primary antibodies used are as follows: anti-dsred 1° antibody ([1:50] Clone Tech, Cat. #632496), rabbit anti-GFAP polyclonal antibody-FITC conjugated (Bioss, Cat# bs-01994-FITC), and rabbit anti-NeuN ([1:100] abcam, Cat. #ab177487). The secondary antibodies used are as follows: Alexa Fluor 594 (1:1,000, Life Tech, Lot #1256153) and Alexa Flour 488 (1:1,000, Thermofisher Scientific, Cat. #A-21206). Only the contrast and brightness were adjusted during imaging.

### Puberty and reproductive phenotype assessment

Males and females were checked for onset of puberty daily starting after weaning at 3 weeks of age. Balanopreputial separation in males was checked by attempting to manually retract the prepuce with gentle pressure. For females, vaginal opening was checked daily [89]. Thereafter, vaginal lavages were collected from experimental mice for at least 3–4 weeks. Cytology of collected cells was examined to assess estrus stages. Predominance of leukocyte cells was taken to indicate a diestrous stage, predominance of nucleated cells a proestrous stage, and predominance of cornified epithelial cells an estrous stage [90, 91]. First estrous was defined as the first day of predominant cornified epithelial cells after the completion of one initial estrous cycle. For fertility studies, adult control IR^loxp^ and IRKO^GFAP^ females 3–4 months old were placed with WT males. Length of time until birth of the first litter and litter size were then determined [2]. The mice were paired for 8 days, and copulatory plugs were observed for evidence of successful mating. After that, mice were separated, and the delivery date was recorded. Similar procedures were used for IR^loxp^ and IRKO^GFAP^ male mice paired with WT females.

### Sexual behavior

IR^loxp^ and IRKO^GFAP^ male mice were paired with WT females on the day the female was in proestrus. IR^loxp^ and IRKO^GFAP^ females were paired with experienced vasectomized males. Mating behavior was captured using infrared cameras (Swann) placed beside individual cages. Mice were placed in the procedure room at 1 PM to acclimate to the new environment and then the lights were turned off at 6 PM to begin the dark phase. After 2 hours in the dark (8 PM), a female in proestrus was introduced into each cage with a single male. Filming began at 8 PM and continued until 2 AM. The following morning, the female mice were checked for copulatory plugs, as previously described [92]. The video files were collected and analyzed for specific hallmarks of female sexual behavior, such as lordosis events and latency to first lordosis, as well as indicators of male sexual interest, such as latency to first mount and number of mounting attempts. A single-blinded rater completed the analysis to ensure consistency and reliability.

### Hormonal assays

Submandibular blood was collected from IR^loxp^ and IRKO^GFAP^ diestrus female and male mice between 8–10 AM in randomly cycling mice to avoid the rise in LH that occurs on proestrus afternoon. LH and FSH levels were measured using multiplex testing performed by the University of Virginia Center for Research in Reproduction (Charlottesville, Virginia). Multiplex LH and FSH levels were measured with intra-assay CV < 20% and reportable range of 0.24–30 ng/ml for LH and 2.4–300 ng/ml for FSH. Female serum estradiol was measured using ELISA (Calbiotech. Spring Valley, California) with sensitivity of 3 pg/ml and intra-assay CV < 10.5%. Male serum testosterone levels were measured by ELISA (Calbiotech. Spring Valley, California) with sensitivity of 0.1 ng/ml and intra-assay CV of 3.17% [93].

### Histology

At 6–7 months of age, adult males and diestrous females were perfused with 10% formalin and organ tissues including the testis or ovary were collected and postfixed immediately in 10% formalin overnight. Next, the tissues were kept in 70% ethanol overnight. Then, tissues were embedded in paraffin, cut into sections, and stained by hematoxylin and eosin [2]. Histological section were visualized via Olympus BX61US microscope (X-cite 120 LED boost EXCELITAS technology) and captured via OlyVia 2.9 software. Ovary sections (4 per mouse) were analyzed by evaluating follicle maturation, including counting the number of primordial, primary, secondary, and preovulatory follicles and corpora lutea. Testes sections were analyzed by evaluating sperm stages, including counting the number of spermatogonium, spermatocytes, spermatid, and spermatozoa. Sperm and follicle counts are reported per seminiferous tubule/ovary cross-section. Only the contrast and brightness were adjusted during imaging.

### Statistical analysis

Data are presented as the mean ± SEM. Two-tailed, unpaired *t* testing was used for comparisons of two groups. One-way ANOVA was used to compare three groups, followed by Bonferroni multiple comparison test. Chi-squared test was used to analyze statistical differences in fertility studies. Data were analyzed using Prism 6 software (GraphPad). *P* < 0.05 was considered statistically significant. The numerical data used in all figures are included in S1 Data.

## Supporting information

S1 Data Excel spreadsheet containing, in separate sheets, the underlying numerical data and statistical analyses for Figs 1, 2, 3, 4, S1, S3, S5, S6, S7, S8 and S9. (XLSX)

S1 Fig Further confirmation of IRKO^GFAP^ model. (A) RTPCR of brain gene expression levels were reported as IR^loxp^ (black bar) and IRKO^GFAP^ (white bar) (*n* = 6–7). Values are expressed as means ± SEM. *P < 0.05 IRKO^GFAP^ versus IR^loxp^ group. The underlying data can be found in S1 Data. (B) PCR gel image showing no differences in Insulin receptor DNA bands between IR^loxp^ and IRKO^GFAP^ in the gonads. GFAP, glial fibrillary acidic protein; IR, insulin receptor; IRKO^GFAP^, astrocyte-specific insulin receptor deletion.
(TIF)

S2 Fig Further validation of immunofluorescence staining of astrocytic Cre-recombination colocalization assay. (A) IF cross section (200 nm and 50 nm) of ARC, AVPV, and cortex for IRKO^GFAP^ stained with GFAP and tdTomato (*n* = 3–4 per group). (B) IF cross section (200 nm and 50 nm) of ARC, AVPV, and cortex for IRKO^GFAP^ stained with NeuN and tdTomato (*n* = 3–4 per group). ARC, arcuate nucleus; AVPV, anteroventral periventricular nucleus; GFAP, glial fibrillary acidic protein; IF, immunofluorescence; IRKO^GFAP^, astrocyte-specific insulin receptor deletion; NeuN.
(TIF)

S3 Fig Further Confirmation of purity of astrocytic FACS isolation. RTPCR of hypothalamic gene expression levels of isolated astrocytes from FACS were reported as RQ (RQ = ^2-ΔΔCt^) for IR^loxp^, IRKO^GFAP^ and IR^loxp^ Brain (*n* = 2 per group). (A) GFAP marker (astrocyte) of FACS sorted cells. (B) MAP-1 marker (neuron) of sorted cells. (C–D) Hes-1 and Hes-5 markers (tanycyte) of sorted cells. (E–F) Cd11b (macrophage) and vWF (endothelial) markers of FACS sorted cells. Astrocytic IR^loxp^ (black bar), astrocytic IRKO^GFAP^ (white bar), and brain (all cells) IR^loxp^ (dashed white bar). Values are expressed as means ± SEM. *P < 0.05 IRKO^GFAP^ versus IR^loxp^ group. The underlying data can be found in S1 Data. Cd11b, cluster of differentiation molecule 11b; FACS, fluorescence-activated cell sorting; GFAP, glial fibrillary acidic protein; Hes, hairy and enhancer of split-1; IR, insulin receptor; IRKO^GFAP^; MAP-1, microtubule associated protein-1; RQ, relative quantification; vWF, Von wellebrand factor gene.
(TIF)

S4 Fig Full representation of western blotting for IR and PGES2. IR, insulin receptor; PGES2, prostaglandin E synthase 2.
(TIF)

S5 Fig IRKO^GFAP^ and IR^loxp^ mice show similar gene expression in bone marrow derived macrophages. (A) RTPCR of astrocytic marker (GFAP) and (B) IR in cultured primary macrophages were reported as RQ (*n* = 3 per group). IR^loxp^ (black bar) and IRKO^GFAP^ (white bar). Values are expressed as means ± SEM. *P < 0.05 IRKO^GFAP^ versus IR^loxp^ group. The underlying data can be found in S1 Data. GFAP, glial fibrillary acidic protein; IR, insulin receptor; IRKO^GFAP^, astrocyte-specific insulin receptor deletion; RQ, relative quantification.
(TIF)

S6 Fig No difference in pubertal timing or adult fertility between control groups. (A–C): Onset of puberty for males and females, balanopreputial separation (*n* = 10–16 per group), vaginal opening (*n* = 9–11 per group), and first estrus (*n* = 10–11 per group). IR^loxp^ (black bar) and Cre (grey bar) (*n* = per group). (D–E) Female adult cyclicity. Estrus cycle length, estrus cycle analysis for which P = predominant nucleated cells (representative of proestrus), E = predominant cornified epithelium cells (representative of estrus), and D = predominant leukocyte cells (representative of metestrus/diestrus) (*n* = 10–11 per group). (F–G) Daily representative of IR^loxp^ estrus stage and IRKO^GFAP^ estrus stage. IR^loxp^ (black line) and Cre (grey line) (*n* = per group). Values are expressed as means ± SEM. *P < 0.05 IRKO^GFAP^ versus the IR^loxp^ group. The underlying data can be found in S1 Data. GFAP, glial fibrillary acidic protein; IR, insulin receptor; IRKO^GFAP^, astrocyte-specific insulin receptor deletion.
(TIF)

S7 Fig No differences in body weight and growth between IRKO^GFAP^ and IR^loxp^ at 3 weeks of age mice. (A–B) Body weight for female (*n* = 10–12 per group) and male mice (*n* = 7–10 per group). (C–D) Body growth for female (*n* = 9–10 per group) and male (*n* = 9–11 per group). IR^loxp^ (black bar) and IRKO^GFAP^ (white bar). Values are expressed as means ± SEM. *P < 0.05 IRKO^GFAP^ versus IR^loxp^ group. The underlying data can be found in S1 Data. GFAP, glial fibrillary acidic protein; IR, insulin receptor; IRKO^GFAP^, astrocyte-specific insulin receptor deletion.
(TIF)

S8 Fig Irregular cyclicity in IRKO^GFAP^ mice. (A–B) Representative cycles of IR^loxp^ (black bar/circle) and IRKO^GFAP^ (white bar/circle). Values are expressed as means ± SEM. *P < 0.05 IRKO^GFAP^ versus IR^loxp^ group. *n* = 10–13 per group. Values are expressed as means ± SEM. *P < 0.05 IRKO^GFAP^ versus IR^loxp^ group. The underlying data can be found in S1 Data. GFAP, glial fibrillary acidic protein; IR, insulin receptor; IRKO^GFAP^, astrocyte-specific insulin receptor deletion.
(TIF)

S9 Fig IRKO^GFAP^ and IR^loxp^ mice show similar sexual behavior around 5 months of age. (A–D): Lordosis events, lordosis quotient, mounting attempts, and latency to first lordosis for females. (E–G) Mounting attempts, latency to first mount, and latency to first intromission for males (*n* = 6–7 per group). IR^loxp^ (black bar) and IRKO^GFAP^ (white bar). Values are expressed as means ± SEM. *P < 0.05 IRKO^GFAP^ versus the IR^loxp^ group. The underlying data can be found in S1 Data. GFAP, glial fibrillary acidic protein; IR, insulin receptor; IRKO^GFAP^, astrocyte-specific insulin receptor deletion.
(TIF)

## Abbreviations

ACSA-1: astrocyte cell surface antigen-1
ARC: arcuate nucleus
APC: allopycocyanin
AVPV: anteroventral periventricular nucleus
FACS: fluorescence-activated cell sorting
FSH: follicle-stimulating hormone
GABA: gamma-amino butyric acid
GFAP: glial fibrillary acidic protein
GnRH: gonadotropin-releasing hormone
HPG: hypothalamic pituitary gonadotropin
IGF: insulin-like growth factor
IR: insulin receptor
IRKO^GFAP^: astrocyte-specific insulin receptor deletion
LH: luteinizing hormone
NeuN: Hexaribonucleotide Binding Protein-3
NIRKO: insulin receptor deletion driven by nestin-cre
PGE2: prostaglandin E2
PGES2: prostaglandin E synthase 2
PND: postnatal day
RFP: red fluorescent protein
RTPCR: reverse transcription polymerase chain reaction
RQ: relative quantification
SYNCAM: synaptic cell adhesion molecule 1
TGF: transforming growth factor
