# Supplementary figures and images for: Ablating astrocyte insulin receptors leads to delayed puberty and hypogonadism in mice
Source: PLoS Biol. 2019 Mar 20;17(3):e3000189. doi: 10.1371/journal.pbio.3000189 (PMC6443191; doi:10.1371/journal.pbio.3000189)

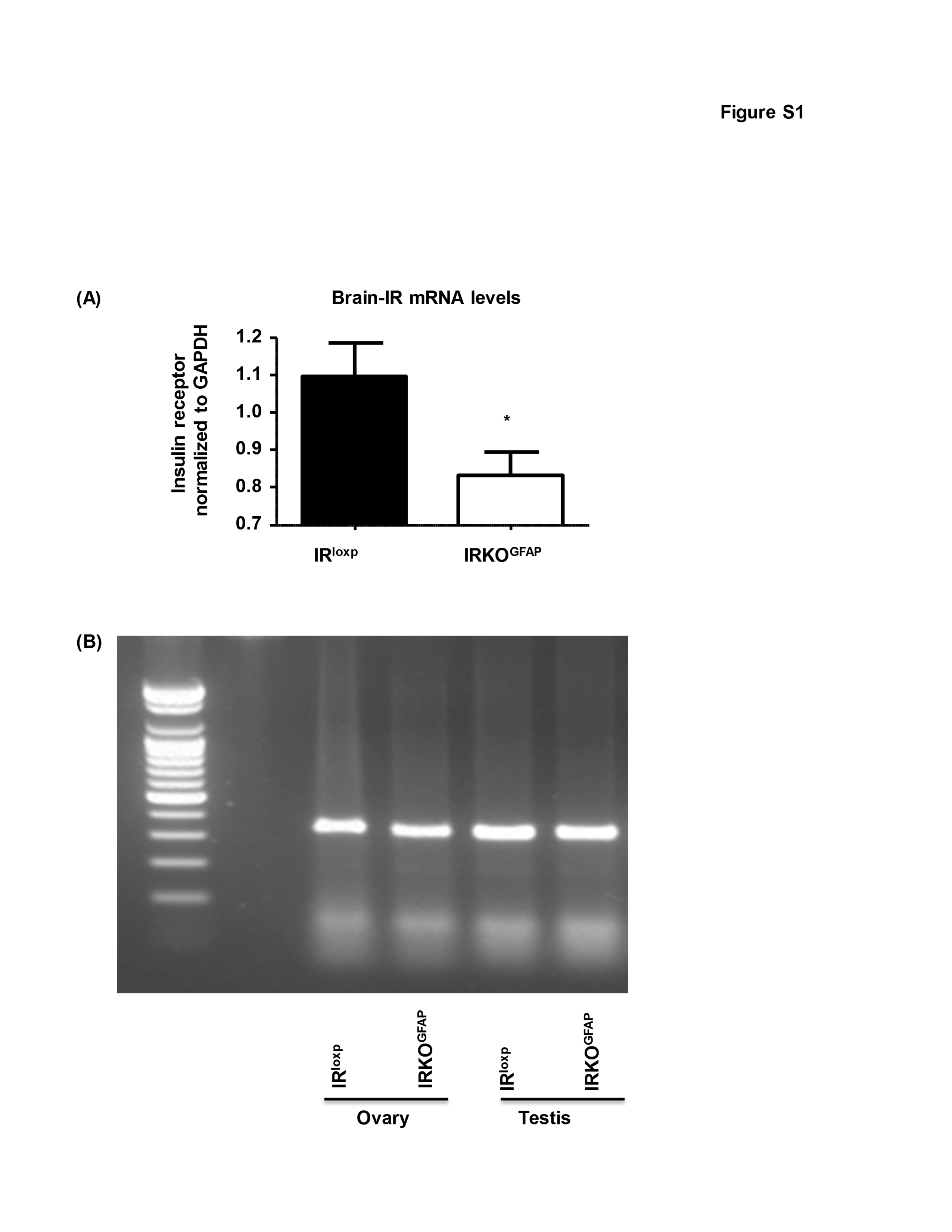

Supplement: S1 Fig — (A) RTPCR of brain gene expression levels were reported as IRloxp (black bar) and IRKOGFAP (white bar) (n = 6–7). Values are expressed as means ± SEM. *P < 0.05 IRKOGFAP versus IRloxp group. The underlying data can be found in S1 Data. (B) PCR gel image showing no differences in Insulin receptor DNA bands between IRloxp and IRKOGFAP in the gonads. GFAP, glial fibrillary acidic protein; IR, insulin receptor; IRKOGFAP, astrocyte-specific insulin receptor deletion. (TIF) [file pbio.3000189.s002.tif]

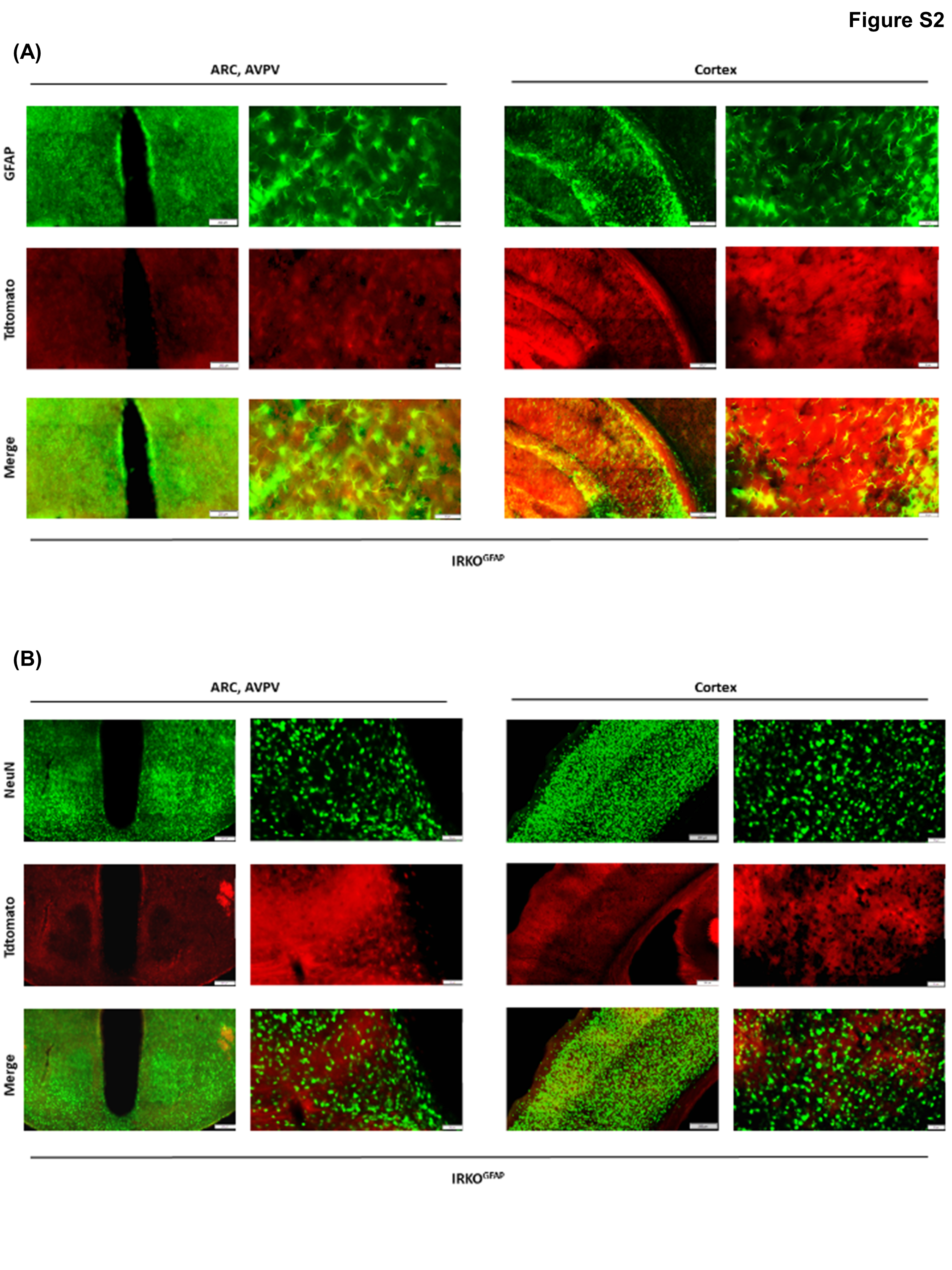

Supplement: S2 Fig — (A) IF cross section (200 nm and 50 nm) of ARC, AVPV, and cortex for IRKOGFAP stained with GFAP and tdTomato (n = 3–4 per group). (B) IF cross section (200 nm and 50 nm) of ARC, AVPV, and cortex for IRKOGFAP stained with NeuN and tdTomato (n = 3–4 per group). ARC, arcuate nucleus; AVPV, anteroventral periventricular nucleus; GFAP, glial fibrillary acidic protein; IF, immunofluorescence; IRKOGFAP, astrocyte-specific insulin receptor deletion; NeuN. (TIF) [file pbio.3000189.s003.tif]

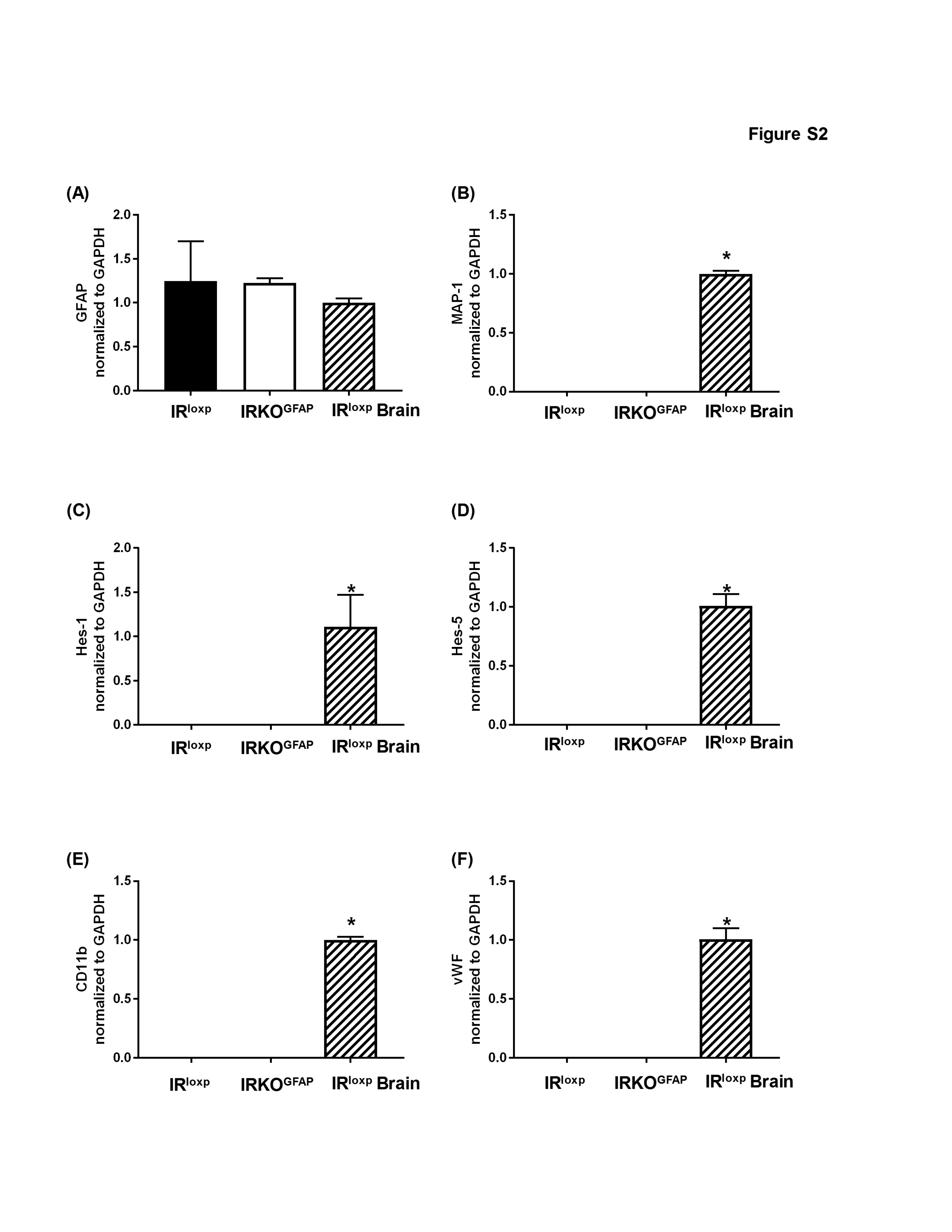

Supplement: S3 Fig — RTPCR of hypothalamic gene expression levels of isolated astrocytes from FACS were reported as RQ (RQ = 2-ΔΔCt) for IRloxp, IRKOGFAP and IRloxp Brain (n = 2 per group). (A) GFAP marker (astrocyte) of FACS sorted cells. (B) MAP-1 marker (neuron) of sorted cells. (C–D) Hes-1 and Hes-5 markers (tanycyte) of sorted cells. (E–F) Cd11b (macrophage) and vWF (endothelial) markers of FACS sorted cells. Astrocytic IRloxp (black bar), astrocytic IRKOGFAP (white bar), and brain (all cells) IRloxp (dashed white bar). Values are expressed as means ± SEM. *P < 0.05 IRKOGFAP versus IRloxp group. The underlying data can be found in S1 Data. Cd11b, cluster of differentiation molecule 11b; FACS, fluorescence-activated cell sorting; GFAP, glial fibrillary acidic protein; Hes, hairy and enhancer of split-1; IR, insulin receptor; IRKOGFAP; MAP-1, microtubule associated protein-1; RQ, relative quantification; vWF, Von wellebrand factor gene. (TIF) [file pbio.3000189.s004.tif]

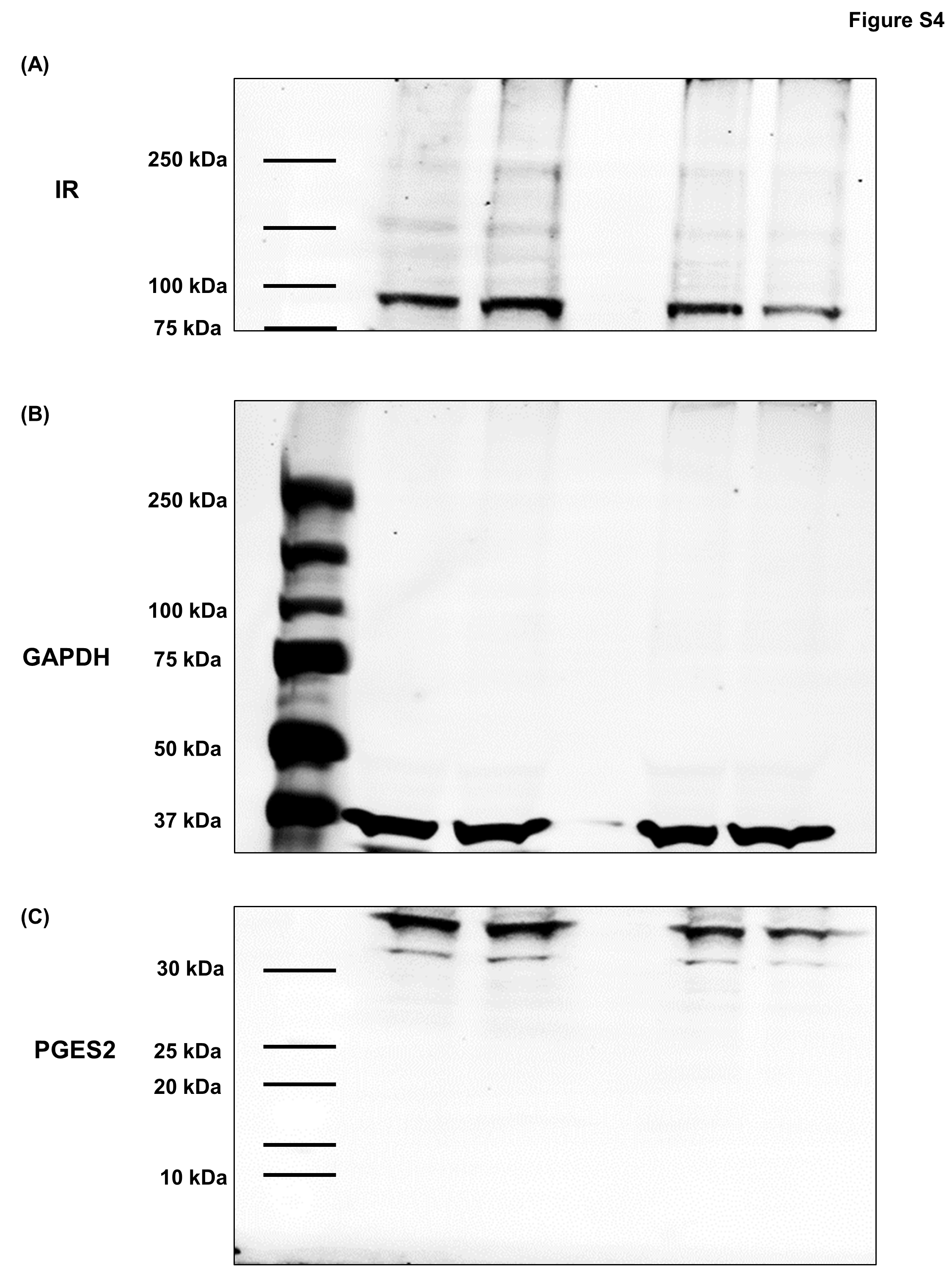

Supplement: S4 Fig — IR, insulin receptor; PGES2, prostaglandin E synthase 2. (TIF) [file pbio.3000189.s005.tif]

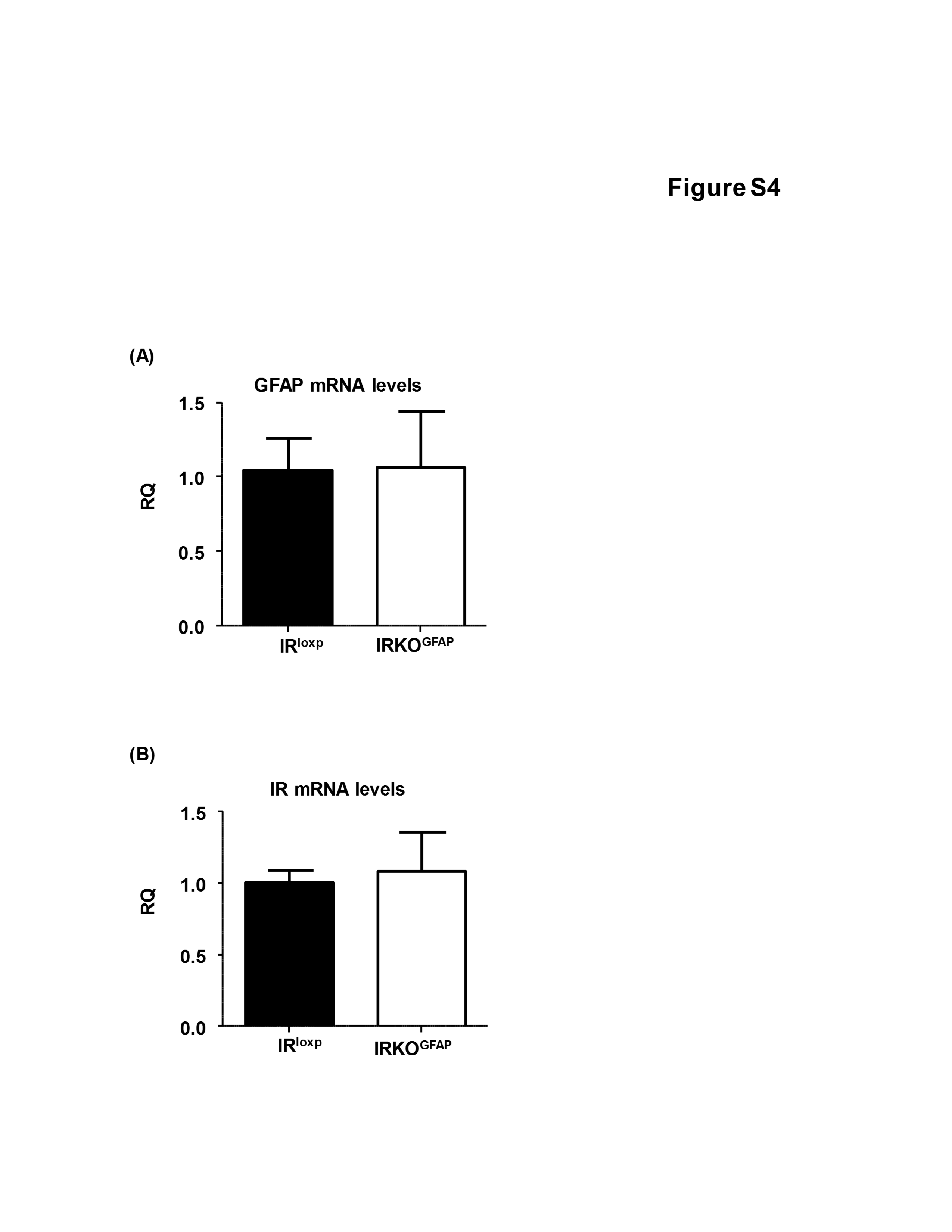

Supplement: S5 Fig — (A) RTPCR of astrocytic marker (GFAP) and (B) IR in cultured primary macrophages were reported as RQ (n = 3 per group). IRloxp (black bar) and IRKOGFAP (white bar). Values are expressed as means ± SEM. *P < 0.05 IRKOGFAP versus IRloxp group. The underlying data can be found in S1 Data. GFAP, glial fibrillary acidic protein; IR, insulin receptor; IRKOGFAP, astrocyte-specific insulin receptor deletion; RQ, relative quantification. (TIF) [file pbio.3000189.s006.tif]

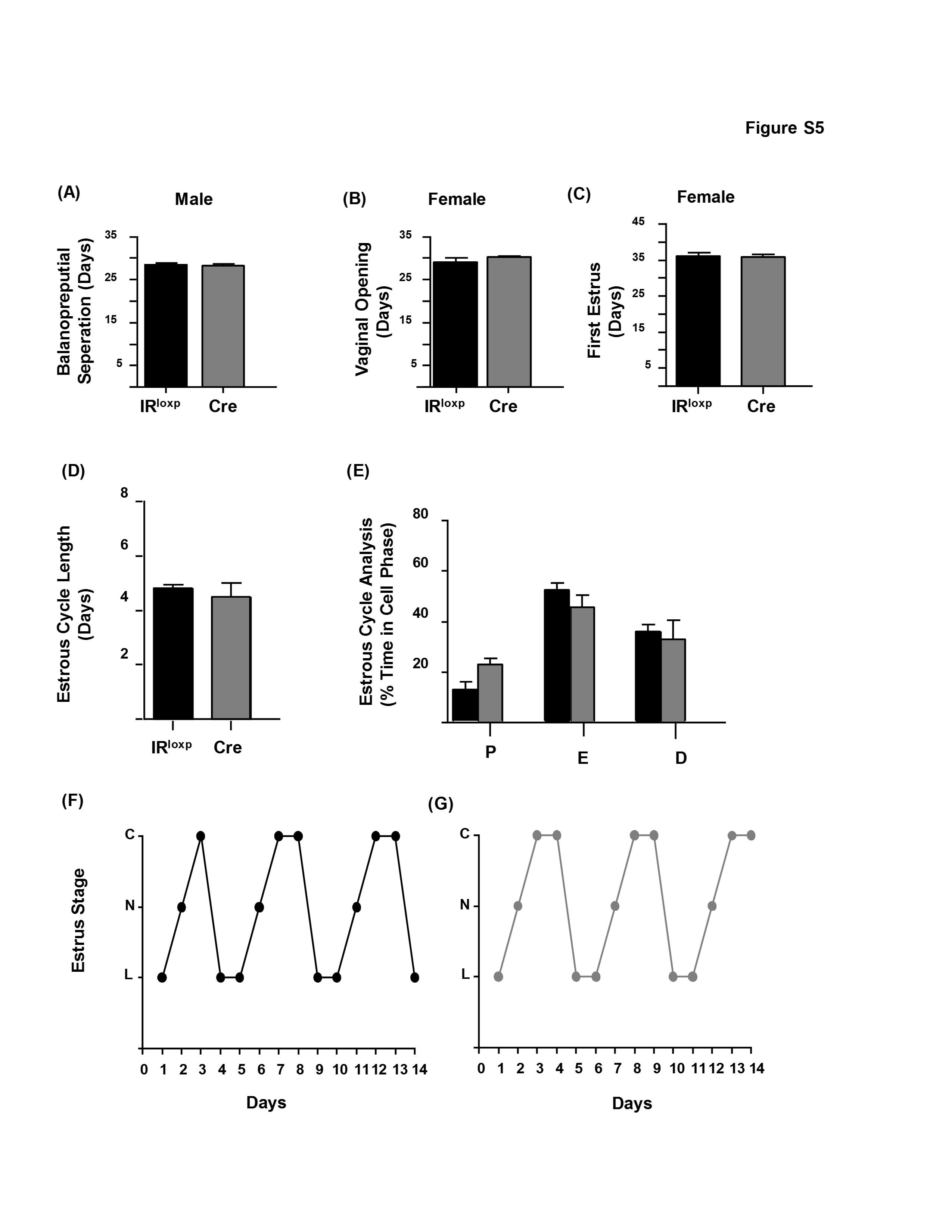

Supplement: S6 Fig — (A–C): Onset of puberty for males and females, balanopreputial separation (n = 10–16 per group), vaginal opening (n = 9–11 per group), and first estrus (n = 10–11 per group). IRloxp (black bar) and Cre (grey bar) (n = per group). (D–E) Female adult cyclicity. Estrus cycle length, estrus cycle analysis for which P = predominant nucleated cells (representative of proestrus), E = predominant cornified epithelium cells (representative of estrus), and D = predominant leukocyte cells (representative of metestrus/diestrus) (n = 10–11 per group). (F–G) Daily representative of IRloxp estrus stage and IRKOGFAP estrus stage. IRloxp (black line) and Cre (grey line) (n = per group). Values are expressed as means ± SEM. *P < 0.05 IRKOGFAP versus the IRloxp group. The underlying data can be found in S1 Data. GFAP, glial fibrillary acidic protein; IR, insulin receptor; IRKOGFAP, astrocyte-specific insulin receptor deletion. (TIF) [file pbio.3000189.s007.tif]

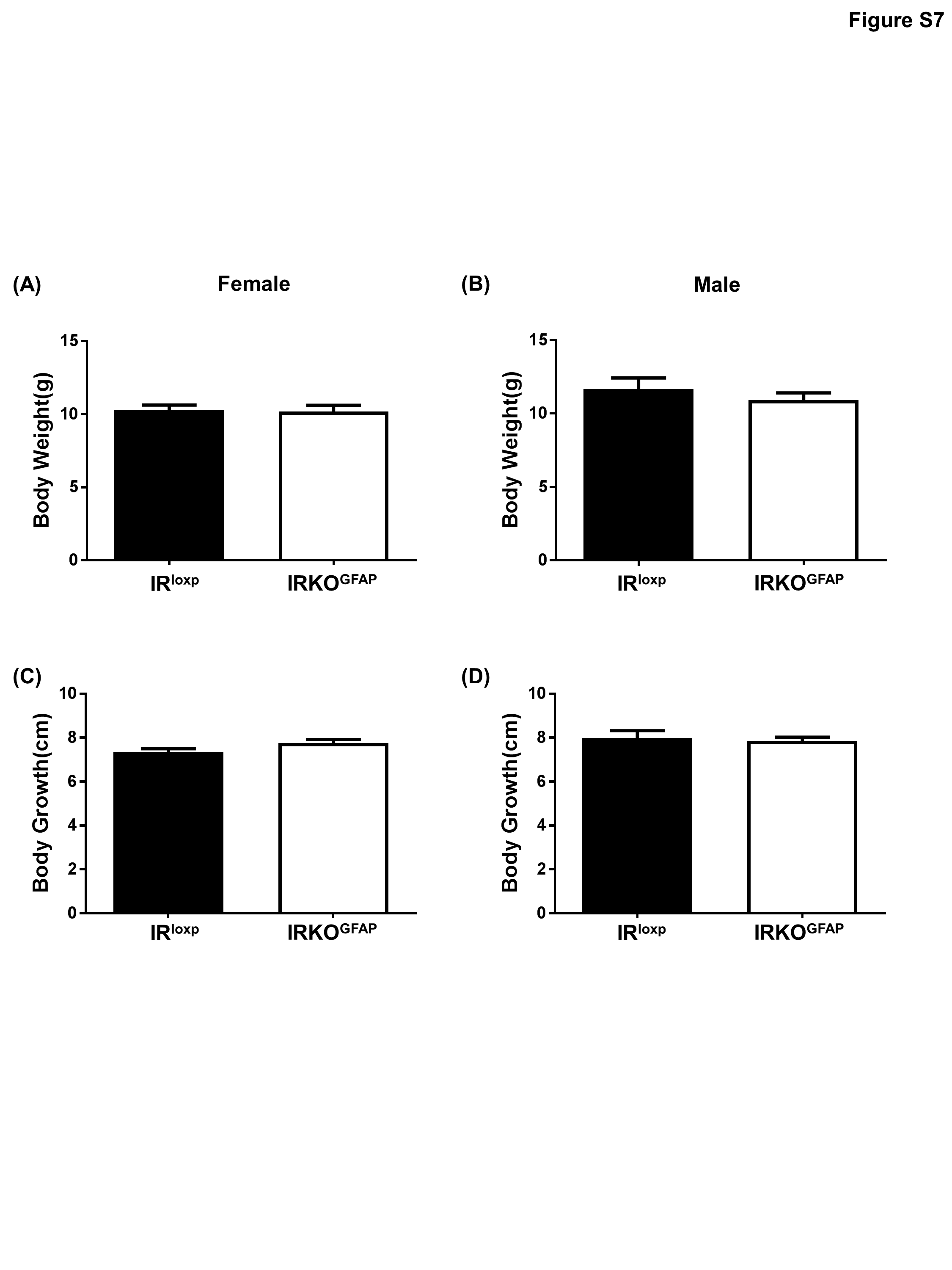

Supplement: S7 Fig — (A–B) Body weight for female (n = 10–12 per group) and male mice (n = 7–10 per group). (C–D) Body growth for female (n = 9–10 per group) and male (n = 9–11 per group). IRloxp (black bar) and IRKOGFAP (white bar). Values are expressed as means ± SEM. *P < 0.05 IRKOGFAP versus IRloxp group. The underlying data can be found in S1 Data. GFAP, glial fibrillary acidic protein; IR, insulin receptor; IRKOGFAP, astrocyte-specific insulin receptor deletion. (TIF) [file pbio.3000189.s008.tif]

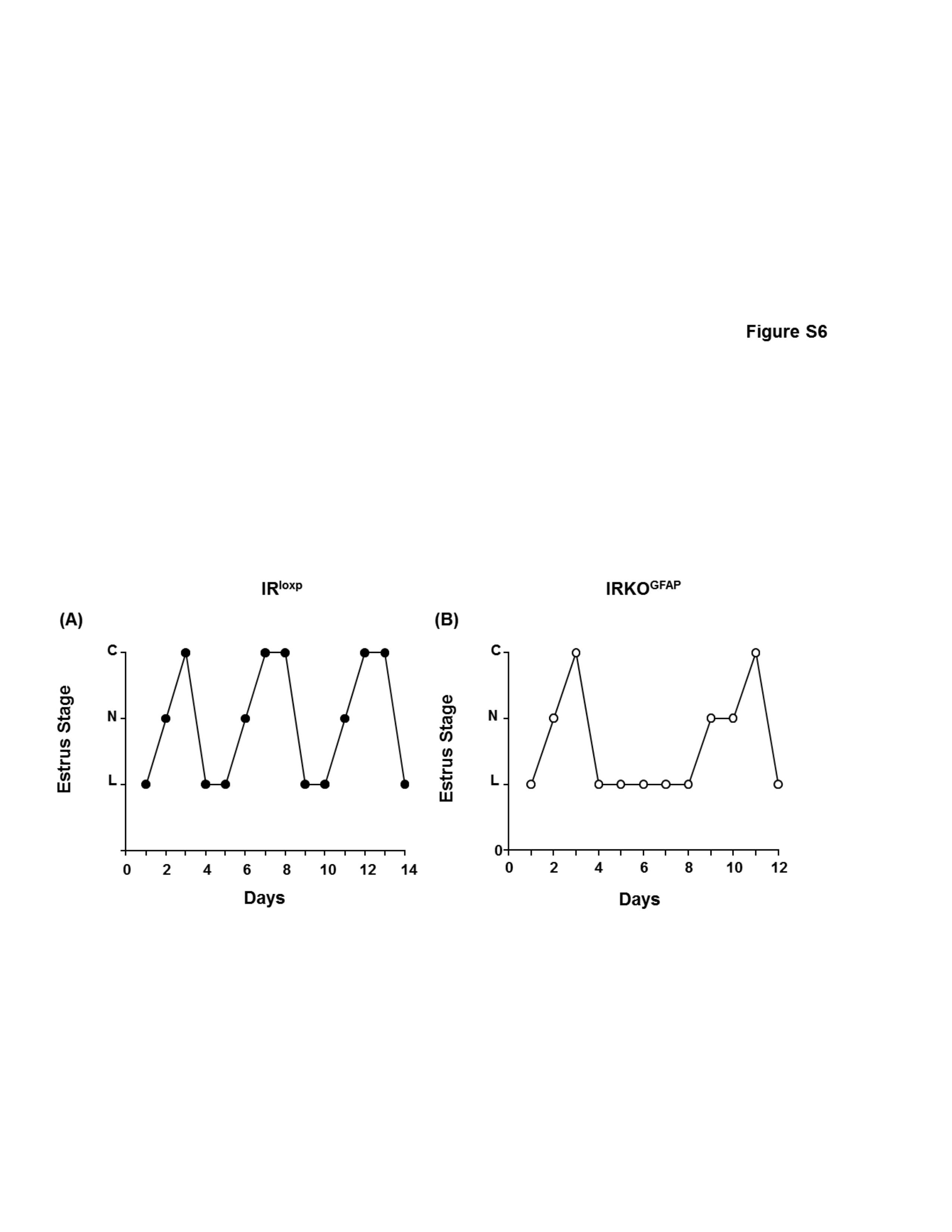

Supplement: S8 Fig — (A–B) Representative cycles of IRloxp (black bar/circle) and IRKOGFAP (white bar/circle). Values are expressed as means ± SEM. *P < 0.05 IRKOGFAP versus IRloxp group. n = 10–13 per group. Values are expressed as means ± SEM. *P < 0.05 IRKOGFAP versus IRloxp group. The underlying data can be found in S1 Data. GFAP, glial fibrillary acidic protein; IR, insulin receptor; IRKOGFAP, astrocyte-specific insulin receptor deletion. (TIF) [file pbio.3000189.s009.tif]

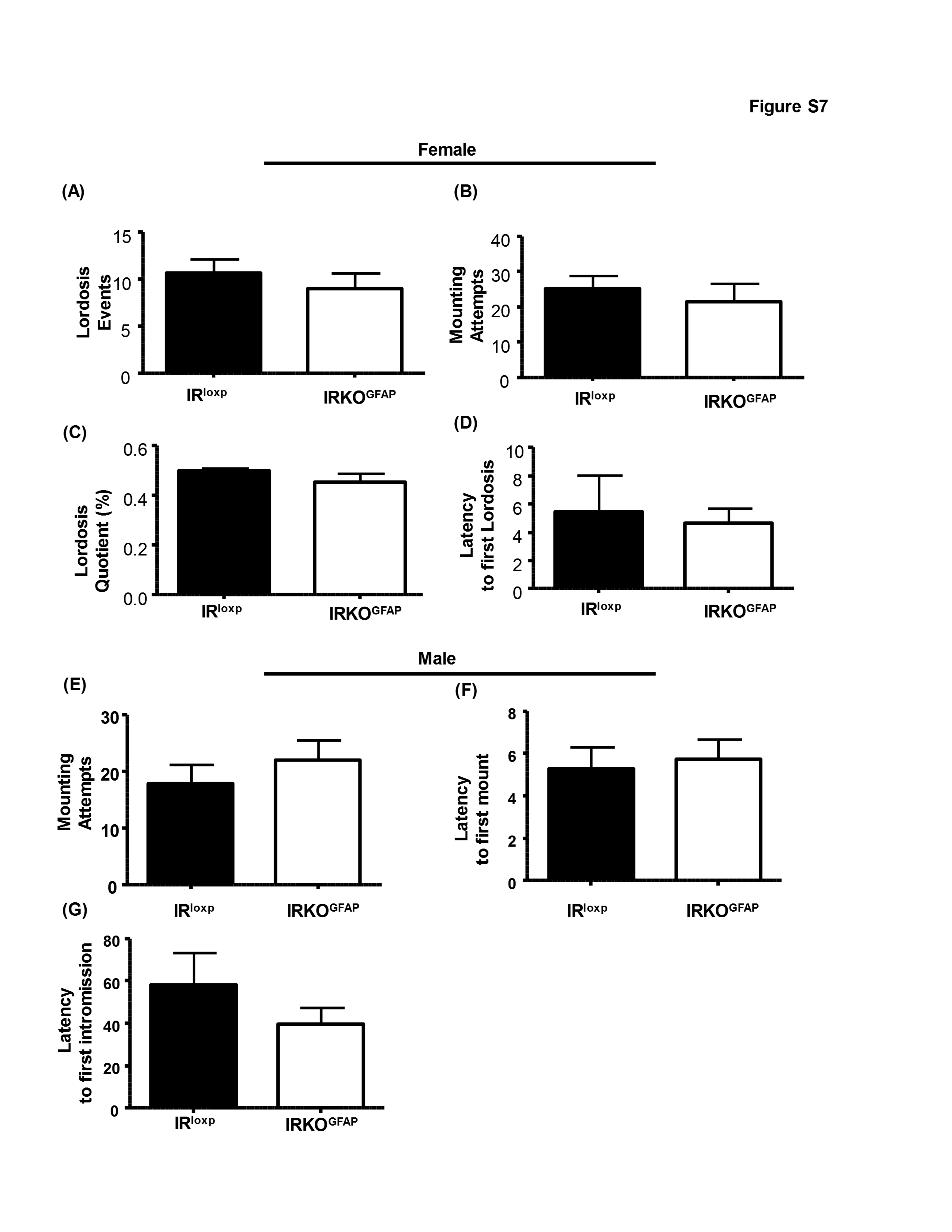

Supplement: S9 Fig — (A–D): Lordosis events, lordosis quotient, mounting attempts, and latency to first lordosis for females. (E–G) Mounting attempts, latency to first mount, and latency to first intromission for males (n = 6–7 per group). IRloxp (black bar) and IRKOGFAP (white bar). Values are expressed as means ± SEM. *P < 0.05 IRKOGFAP versus the IRloxp group. The underlying data can be found in S1 Data. GFAP, glial fibrillary acidic protein; IR, insulin receptor; IRKOGFAP, astrocyte-specific insulin receptor deletion. (TIF) [file pbio.3000189.s010.tif]
